# Assessment of disability in the older adults using electronic health record–based data: a machine learning approach

**DOI:** 10.3389/fpubh.2026.1823727

**Published:** 2026-05-01

**Authors:** Guangpeng Chen, Shunyu Wang, Li Luo

**Affiliations:** 1 School of Public Health, Fudan University, Shanghai, China; 2 School of Biomedical Engineering, Faculty of Medicine, Dalian University of Technology, Dalian, China; 3 Department of International Medical, The Second Affiliated Hospital of Dalian Medical University, Dalian, China

**Keywords:** disability assessment, older adults, electronic health record, machine learning, Barthel Index

## Abstract

**Background:**

Global population aging is accelerating, and China faces a significant challenge with “aging before affluent.” The disabled older adults population in China is projected to reach 58 million by 2050. Early identification of disability risk is essential for optimizing healthcare resource allocation and improving long-term care systems.

**Objective:**

This study aimed to develop and validate machine learning models using comprehensive electronic medical record (EMR) data to predict four distinct levels of disability in older adults inpatients.

**Methods:**

Data from 523 patients (age ≥ 60) were retrospectively collected from two tertiary hospitals in Northeast China. Disability was categorized into four levels (A–D) based on the Barthel Index (BI). Feature selection was performed using LASSO regression with 10-fold cross-validation. Six algorithms—Logistic Regression (LOG), Random Forest (RF), Gradient Boosting Machine (GBM), XGBoost, AdaBoost, and Support Vector Machine (SVM)—were evaluated. SHapley Additive exPlanations (SHAP) was employed to interpret model decisions.

**Results:**

Seventeen critical predictors were identified, including age, appendicular skeletal muscle mass index (ASMI), phase angle, and various inflammatory markers. The LOG and XGBoost models demonstrated the best performance (AUC = 0.83; Accuracy = 0.91). Ten-fold cross-validation confirmed stable model. SHAP analysis indicated that neurological comorbidities, muscle mass indicators, and nutritional-inflammatory status were the primary drivers of disability risk.

**Conclusion:**

EMR-based machine learning models, particularly XGBoost, provide a robust and interpretable tool for early disability risk stratification, supporting clinical decision-making.

## Introduction

Global aging is accelerating at an unprecedented rate, characterized by significant regional disparities. According to the World Population Prospects 2024, individuals aged 65 and over accounted for approximately 11% of the global population in 2024; this figure is projected to reach 16.3% by 2050 and exceed 21% by the end of this century (1). This demographic shift is driven by declining fertility rates and increased life expectancy resulting from medical advancements and improved public health infrastructure. However, these trends also lead to expanded pension deficits, surging expenditures for medical and long-term care, a shrinking labor supply, and downward pressure on potential economic growth.

A critical challenge posed by this aging population is the rising prevalence of functional impairment and disability among the older adults. Disability—defined as the decline or loss of Basic Activities of Daily Living (ADL) and Instrumental Activities of Daily Living (IADL) due to age, disease, or injury—severely compromises independent living. Standardized assessment tools, such as the ADL scale, IADL scale, WHO Disability Assessment Schedule (WHODAS 2.0), and the Functional Independence Measure (FIM), are currently the primary instruments for evaluating these states. Among these, the Barthel Index (BI) remains a gold standard for functional evaluation in clinical settings (2). ADL assessment is of particular value in prognostic determination; compared to certain clinical indicators, ADL status offers a more efficient and practical tool for predicting survival outcomes and facilitating risk stratification in primary geriatric health management (3).

Globally, the disability rate among individuals aged 60 and over is approximately 38.1%, with significant regional variations: approximately 20% in developed countries and nearly 50% in Africa. Disability is highly correlated with chronic comorbidities, sarcopenia, and cognitive impairment (4). China, currently hosting the world’s largest older adults population with 280 million people aged 60 and over (19.8%), has officially entered a stage of deep aging. Projections indicate that the number of disabled older adults in China will rise from 35 million in 2024 to 58 million by 2050, with disability rates reaching 40% among those over 80 years old (5). Furthermore, significant disparities exist across urban–rural divides and gender, with rural residents and women facing higher risks. As the aging crisis intensifies, the accurate identification of high-risk inpatients becomes paramount for implementing targeted preventive measures (6).

Despite its importance, traditional disability assessment remains highly dependent on manually trained professionals, a process that is both time-consuming and costly. There is an urgent need for automated, data-driven prediction tools to identify patients requiring prioritized evaluation and to guide the rational allocation of medical resources.

Electronic Medical Records (EMRs), which originated as academic pilots in the 1960s, have evolved into the core infrastructure for clinical practice, management, and research (7). With global adoption rates exceeding 85%, EMRs provide a continuous and multidimensional record of vital signs, laboratory results, diagnostic information, and health-related behaviors (8). These systems not only enhance diagnostic efficiency and medication safety but have also been shown to reduce adverse events by approximately 25% (9). The widespread integration of EMRs enables the use of machine learning to construct disability prediction models based on real-world clinical data (10). Recent studies have highlighted the increasing potential of machine learning approaches for functional status assessment and health outcome prediction in older adults (11–13). While previous studies have utilized traditional regression models often limited to binary outcomes, there remains a significant research gap regarding multi-class disability modeling and systematic algorithmic comparisons (14–16).

This study aims to fill this gap by utilizing EMR data from two tertiary hospitals in Dalian, China, to construct a four-level disability prediction model (none, mild, moderate, and severe). By employing LASSO regression for feature selection and comparing six mainstream machine learning algorithms, we utilized SHAP analysis to reveal the significance and directionality of various clinical features across different disability stages.

## Methods

**Figure 1:**
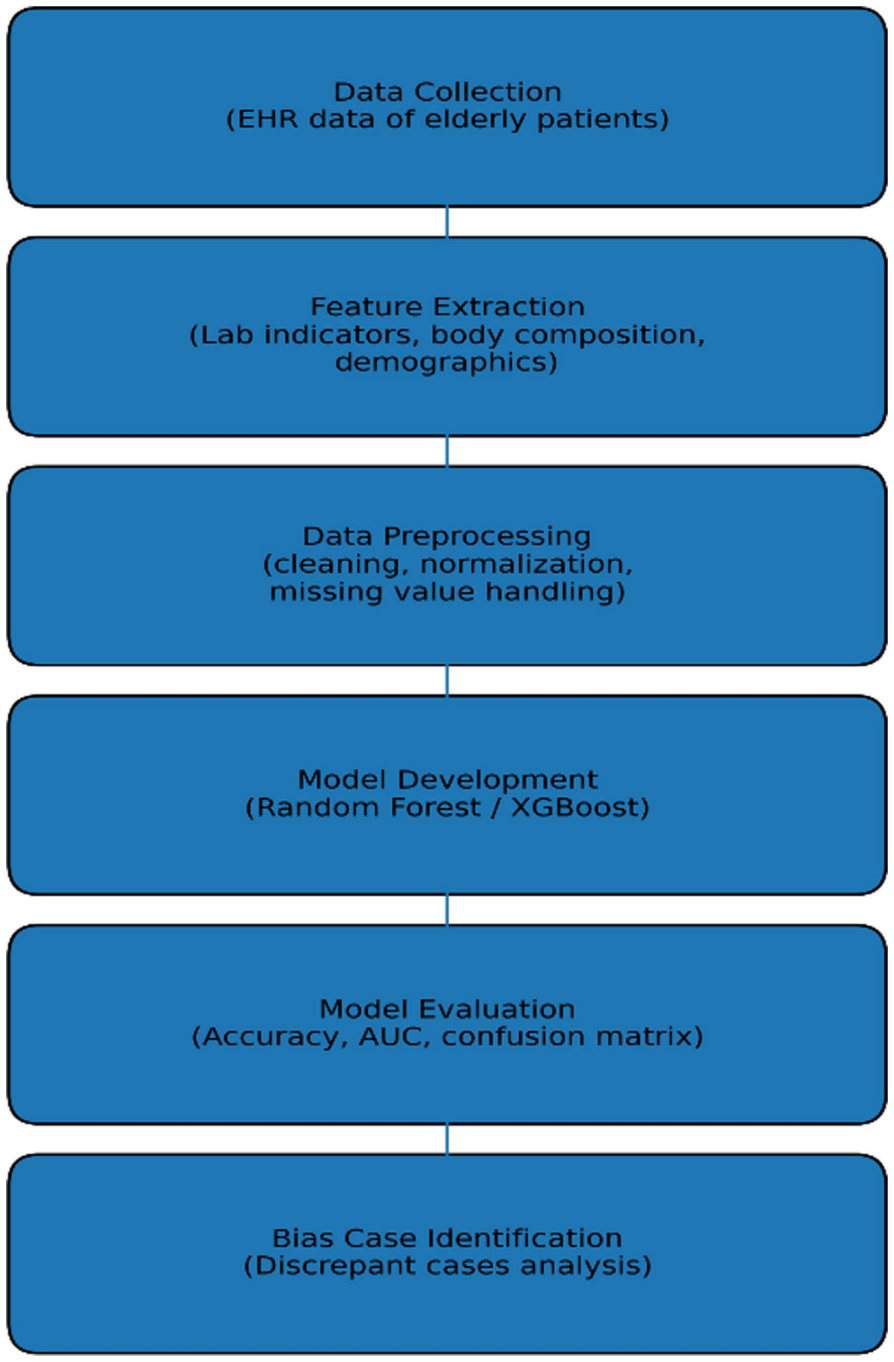
Research workflow of the machine learning–based disability assessment study.

The overall workflow of the study is illustrated in Figure 1. The workflow illustrates the overall process of the study, including electronic health record (EHR) data collection, feature extraction, data preprocessing, machine learning model development, model evaluation, and identification of discrepant cases for bias analysis.

### Data source and study population

This retrospective study analyzed electronic medical record (EMR) data from two tertiary hospitals in Dalian, Liaoning Province, China. Each record represented a single inpatient admission, with key fields including unique patient ID (medical record number), age, gender, admission date, date of birth, medical history, and clinical findings (past medical history, present illness history, chief complaint, physical examination, auxiliary exams, diagnosis, and treatment plans). Data collection spanned January 1, 2024, to June 1, 2025, with 523 cases ultimately included after applying inclusion criteria (age ≥ 60 years; at least one complete disability assessment, hospitalization record, and baseline laboratory examination) and exclusion criteria (data missing rate > 30%). The study included 523 participants with a weighted mean age of 70.32 years, ranging from 60.0 to 93.0 years. The study received Institutional Ethics Committee approval (Ref: KY2025-738-01), with all patients providing informed consent for anonymous data use in research.

### Inclusion and exclusion criteria inclusion criteria

Age ≥ 60 years; At least one complete record of disability level assessment; At least one hospitalization record; At least one complete baseline laboratory examination.

Exclusion Criteria: A data missing rate exceeding 30% for the overall individual record.

### Baseline variables and pre-selected predictive factors

Baseline information includes structural data and laboratory indicators extracted from the EMRs. Specifically, these include clinical features such as Supplementary Table S1 primary disease categories, Supplementary Table S2 Socio-demographic characteristics, Supplementary Table S3 Body composition-related indicators, Supplementary Table S4 Laboratory indicators.

### Outcome definition: four-level disability grading

Disability was assessed using the original Barthel Index (BI) (17), a validated tool for geriatric inpatients with high inter-rater reliability (ICC = 0.95) and test–retest reliability (ICC = 0.93) (18, 19). BI scores categorized disability into four levels: Group A (No Disability); Group B (Mild); Group C (Moderate); and Group D (Severe). Baseline variables encompassed structured data and laboratory indicators, including primary disease categories, socio-demographic characteristics, body composition indicators, and laboratory measures.

### Data preprocessing and training/testing split

Subjects with > 30% missingness were removed, continuous variable outliers were truncated via the interquartile range (IQR) method, and categorical variables underwent one-hot encoding. The dataset was split using stratified hold-out sampling by disability level (training: 70%, *n* = 366; testing: 30%, *n* = 157), with Synthetic Minority Over-sampling Technique (SMOTE) applied to the training set to address class imbalance, perform in Tables 1, 2. LASSO multi-class logistic regression was conducted to select predictors for model construction. The models built in current study includes: Logistic Regression (LOG), XGBoost, Support Vector Machine (SVM), AdaBoost, Gradient Boosting Machine (GBM), and Random Forest (RF). Evaluation metrics include AUC, Accuracy, Cohen’s kappa, macro F1-score, sensitivity, and specificity. For model interpretability, SHAP (SHapley Additive exPlanations) analysis was conducted on the best-performing model.

**Table 1:** Original training and test set grouping.

Group	Training set	Test set	Total
A	231	99	330
B	86	37	123
C	28	12	40
D	21	9	30

**Table 2:** Training set after SMOTE.

Group	Training set	SMOTE
A	231	231
B	76	173
C	28	76
D	21	61

### Feature selection and modeling

**Figure 2:**
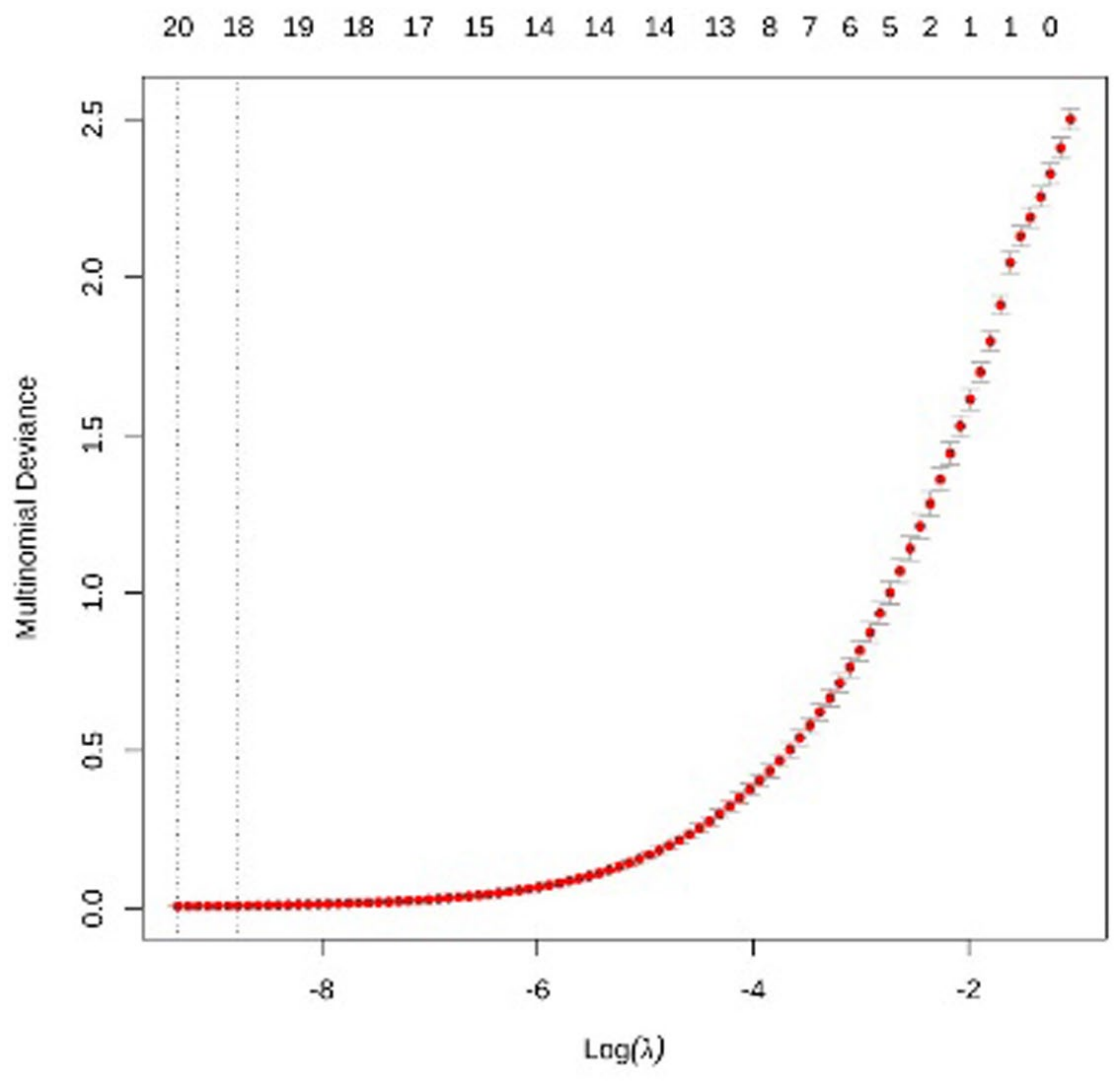
10-fold cross-validation process used to select the optimal penalty parameter lambda. The *x*-axis represents the log-transformed penalty parameter [Log(lambda)], and the *y*-axis represents the multinomial deviance. The red dots indicate the mean deviance values obtained for each lambda, with the error bars representing the standard error. Two vertical dashed lines designate the lambda values associated with the minimum deviance and the one-standard-error rule. The optimal parameter was determined to be lambda = 0.00018, where the multinomial deviance reached its minimum. At this specific lambda value, the regression coefficients of non-influential variables were shrunk to zero, while 17 core variables were retained as key predictors.

**Figure 3:**
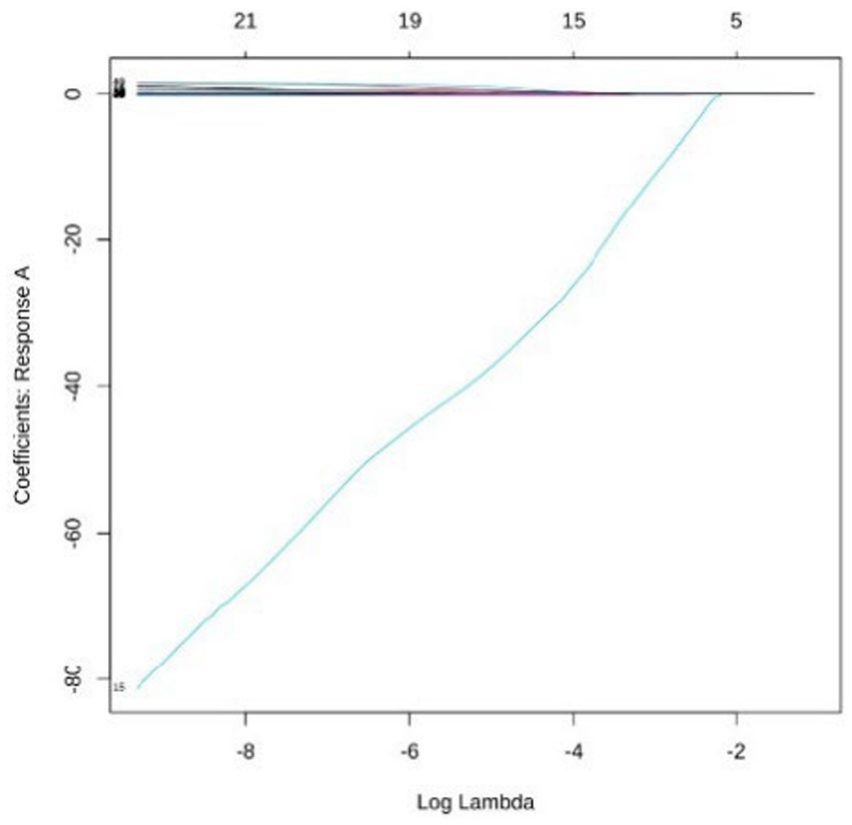
Analysis of coefficient dynamics across disability levels group A. The coefficient path for response A illustrates variables associated with the absence of disability. A dominant feature is observed with a large negative coefficient, suggesting a strong protective factor that remains non-zero even as the penalty is significantly increased.

**Figure 4:**
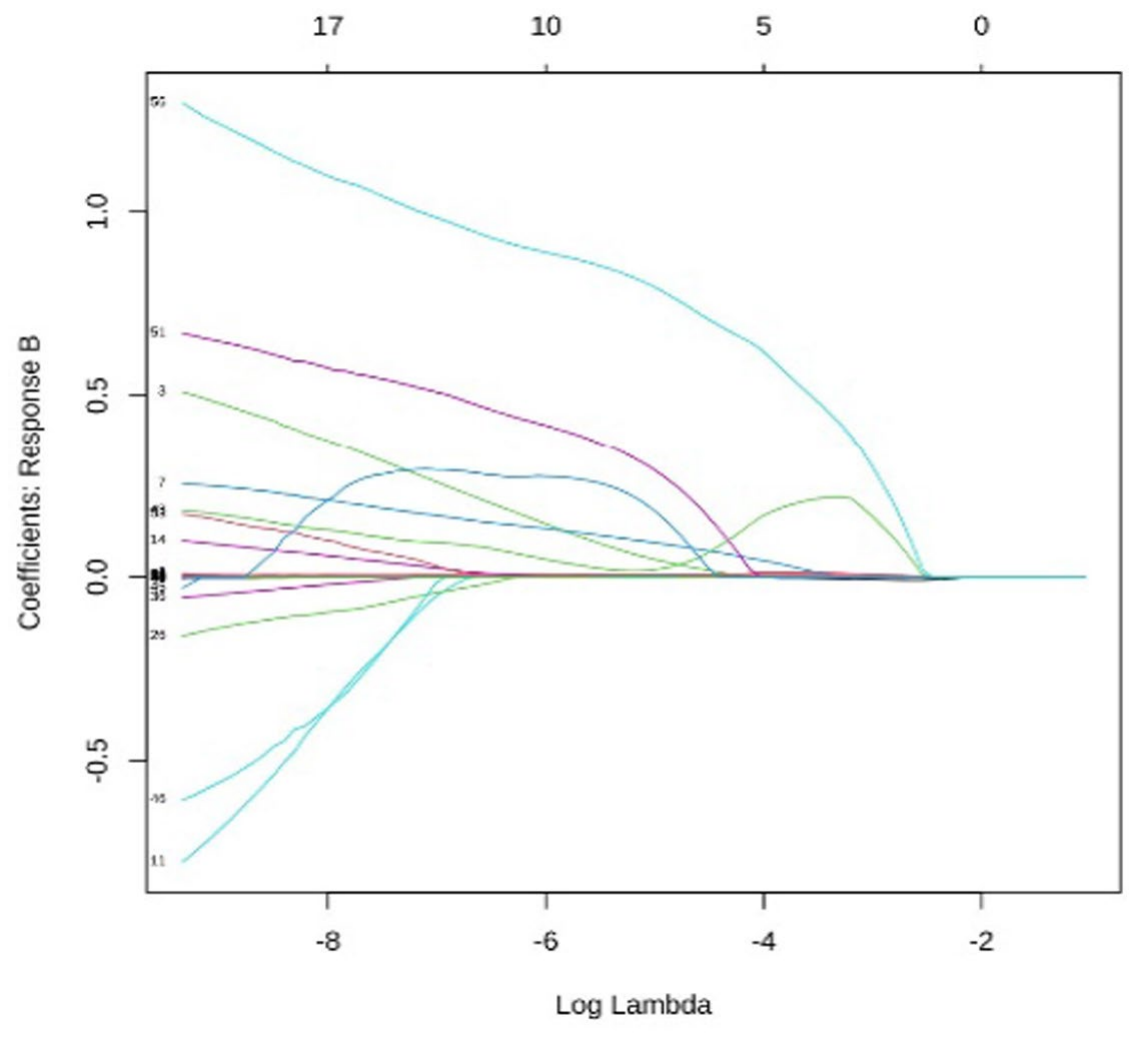
Analysis of coefficient dynamics across disability levels group B: the paths for mild disability display a diverse set of predictors, including both positive and negative coefficients. This complexity reflects the transitional nature of mild functional decline, where multiple clinical and physiological factors begin to exert influence.

**Figure 5:**
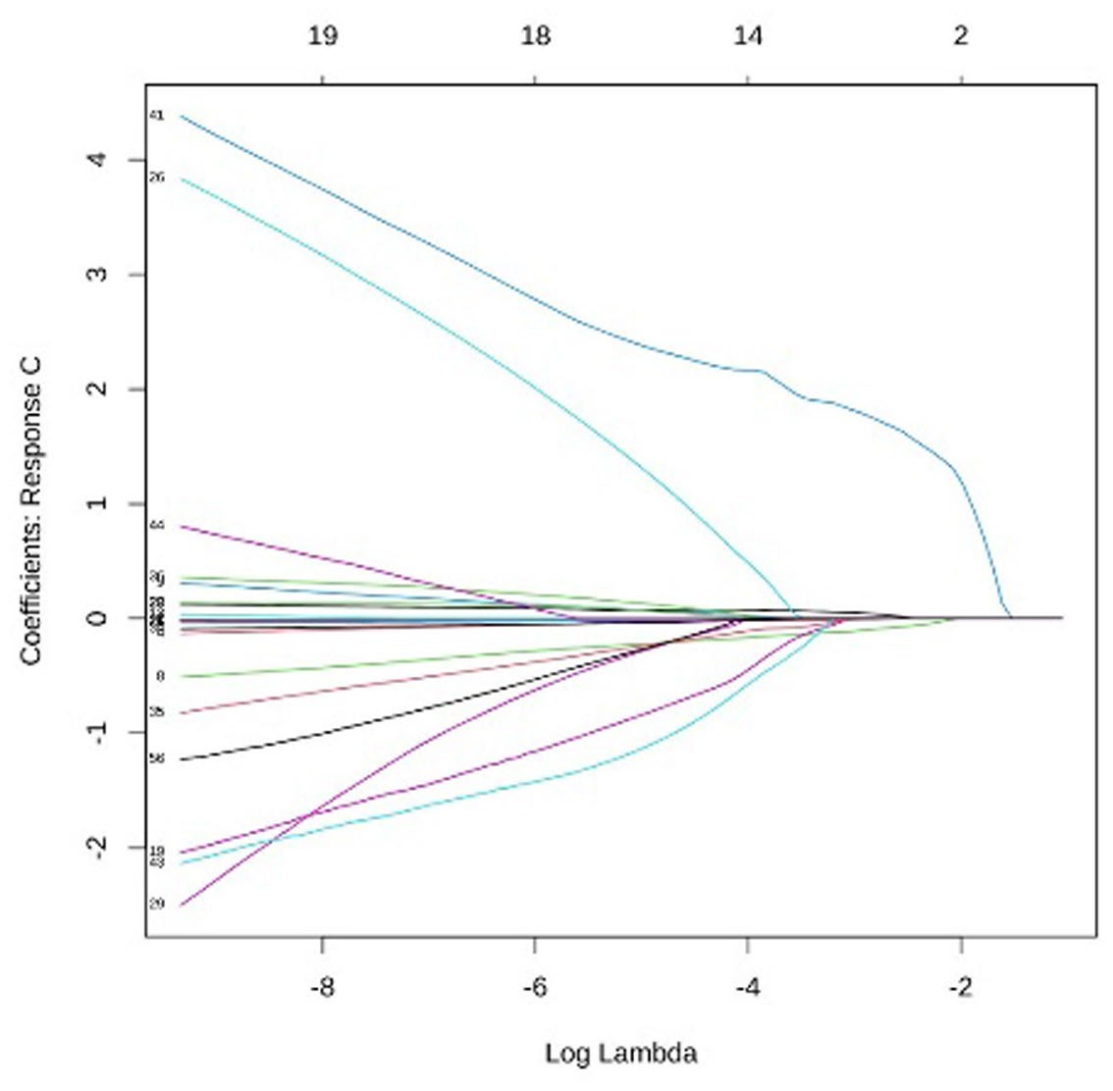
Analysis of coefficient dynamics across disability levels group C: for moderate disability, the paths show a higher density of influential variables at lower lambda values. Several predictors maintain stable non-zero coefficients across a wide range of log lambda, indicating their robustness in distinguishing moderate impairment from other levels.

**Figure 6:**
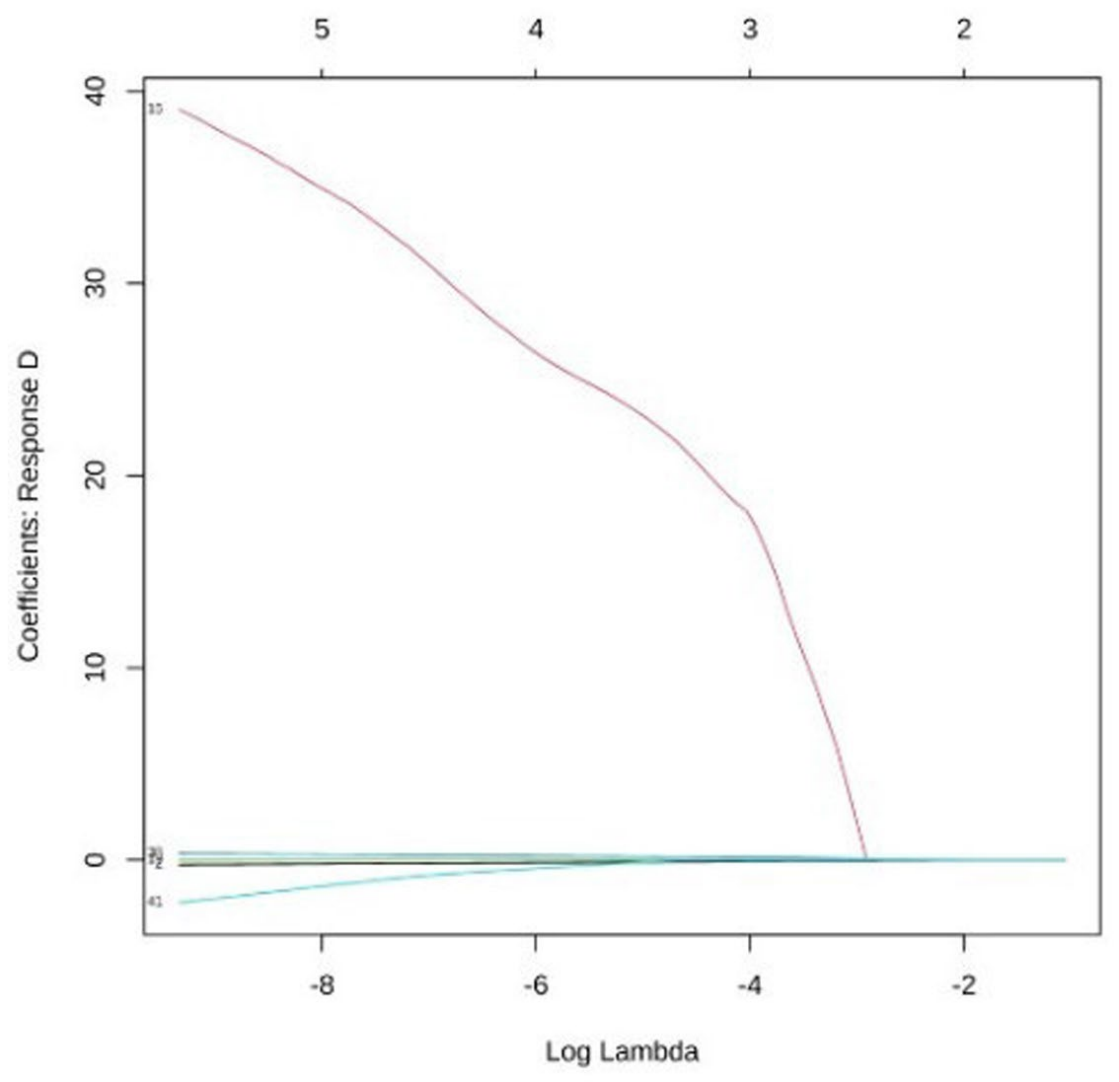
Analysis of coefficient dynamics across disability levels group D: the coefficient path for severe disability is characterized by a single predictor with a markedly high positive coefficient. This variable shows exceptional persistence against the shrinkage penalty, identifying it as a primary diagnostic marker for the most severe functional loss.

LASSO multi-class logistic regression was employed for feature selection to prevent overfitting and enhance interpretability. Utilizing 10-fold cross-validation, the optimal penalty parameter was determined to be lambda = 0.00018. The dataset was split using a hold-out strategy into a training set (70%, *n* = 366) and a testing set (30%, *n* = 157). To address class imbalance in Groups C and D, the Synthetic Minority Oversampling Technique (SMOTE) was applied to the training set. Full details are available in Figures 2–6.

At lambda = 0.00018, 17 core predictors were retained: age, appendicular skeletal muscle mass index (ASMI), phase angle, BMI, visceral fat area, extracellular water ratio, hs-CRP, hemoglobin, albumin, prealbumin, Vitamin D, HbA1c, eGFR, homocysteine, COPD, stroke, and dementia syndrome.

### Descriptive statistics of variables selected after lasso screening

Table 3 presents the descriptive statistics of the 17 selected variables across the four disability groups.

**Table 3:** Descriptive statistics of selected variables across disability groups (A–D).

Variable	A	B	C	D	*p*-value
Demographics and anthropometrics					
Age (years)	68.0 (6.6)	71.5 (6.2)	78.5 (5.7)	80.2 (4.3)	< 0.001
ASMI (kg/m^2)	7.25 (0.85)	6.85 (0.80)	6.40 (0.75)	5.95 (0.70)	< 0.001
Phase angle (^circ)	6.1 (0.9)	5.5 (0.8)	4.9 (0.7)	4.3 (0.6)	< 0.001
BMI (kg/m^2)	24.0 (3.0)	23.9 (2.9)	23.7 (2.8)	21.9 (2.7)	0.021
Visceral fat area (cm^2)	95.5 (28.0)	105.0 (30.5)	115.0 (32.1)	118.0 (34.2)	< 0.001
ECW Ratio (%)	38.2 (1.5)	39.0 (1.6)	39.8 (1.7)	40.5 (1.8)	< 0.001
Biochemical indicators					
hs-CRP (mg/L)	1.5 (0.8)	2.8 (1.5)	5.2 (2.1)	8.5 (3.5)	< 0.001
Hemoglobin (g/L)	139 (17.4)	126 (18.7)	116 (27.7)	105 (15.2)	< 0.001
Albumin (g/L)	42.5 (4.4)	38.2 (6.8)	35.9 (3.7)	32.1 (5.2)	< 0.001
Prealbumin (mg/L)	287 (41.9)	264 (41.2)	252 (23.7)	195 (30.5)	< 0.001
Vitamin D (ng/mL)	20.4 (6.9)	22.8 (7.5)	18.5 (5.2)	15.2 (4.1)	< 0.001
HbA1c (%)	6.5 (1.5)	7.92 (1.80)	8.16 (1.74)	7.50 (1.20)	0.004
eGFR (mL/min/1.73 m^2)	84.6 (10.5)	83.2 (13.6)	75.4 (12.1)	68.5 (15.3)	< 0.001
Homocysteine (mu mol/L)	12.3 (4.3)	11.1 (3.1)	13.5 (1.8)	16.8 (5.5)	< 0.001
Disease status [*n* (%)]					
COPD	60 (18.2%)	16 (13.0%)	11 (27.5%)	12 (40.0%)	< 0.001
Stroke	109 (33.0%)	59 (48.0%)	5 (12.5%)	4 (13.3%)	< 0.001
Dementia syndrome	37 (11.2%)	19 (1.5%)	10 (2.5%)	29 (9.7%)	< 0.001

## Results

### Machine learning models and interpretation

**Figure 7:**
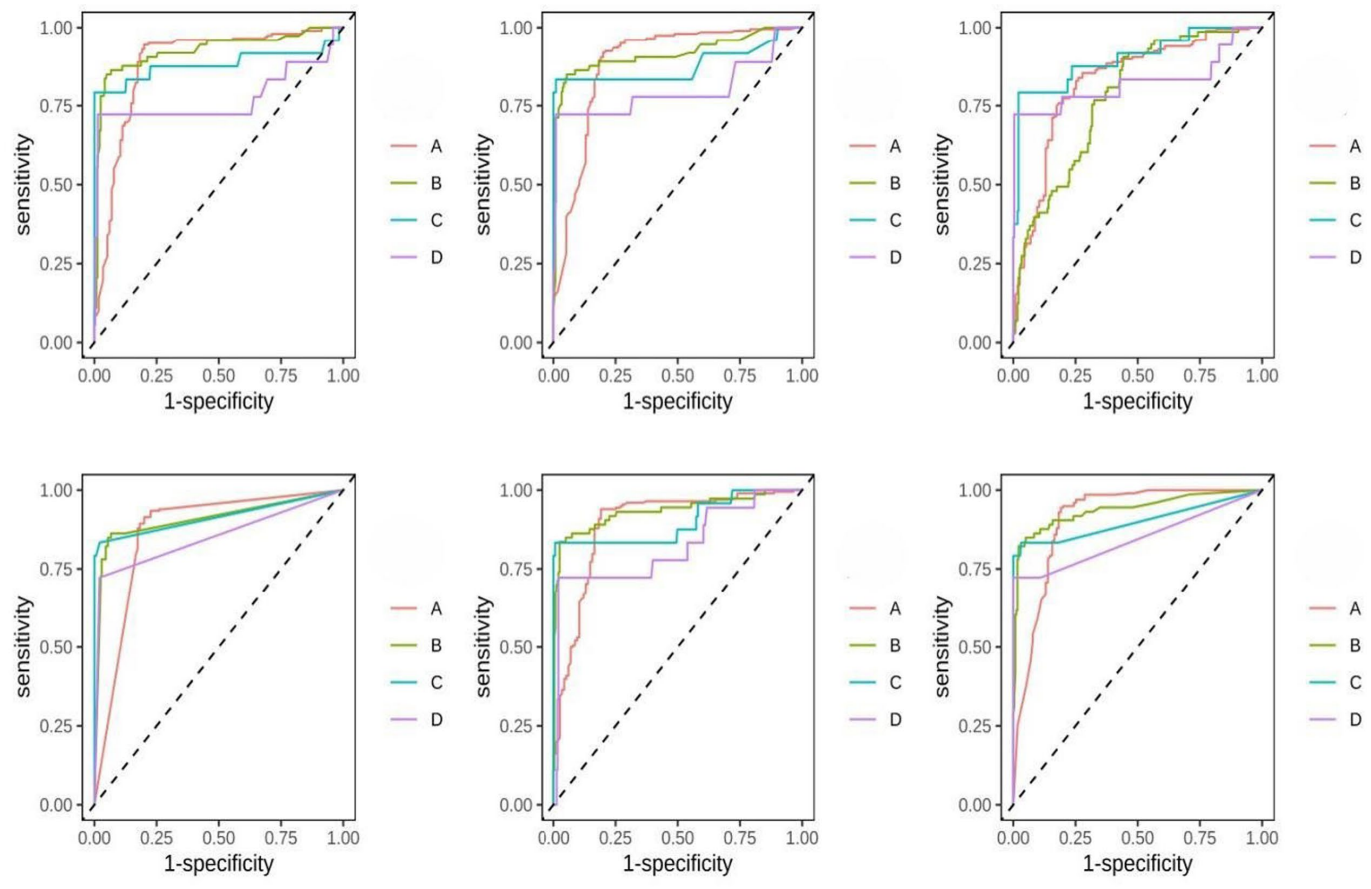
The predictive performance of six different models: the overall model performance evaluation displays the receiver operating characteristic (ROC) curves of six machine learning algorithms on an independent test set for four levels of disability (Groups A–D). The experimental results show that logistic regression (LOG) and extreme gradient boosting (XGBoost) performed best in terms of overall predictive ability, with both achieving an area under the receiver operating characteristic curve (AUC) of 0.83 and an overall accuracy of 0.91. Furthermore, both LOG and XGBoost had a Cohen’s kappa coefficient of 0.80, indicating a high degree of agreement between the model’s predictions and the actual disability levels.

Six algorithms were employed to predict the four disability levels: Logistic Regression (LOG), XGBoost, Support Vector Machine (SVM), AdaBoost, Gradient Boosting Machine (GBM), and Random Forest (RF). Model performance was evaluated using AUC, Accuracy, Cohen’s kappa, macro F1-score, sensitivity, and specificity. For model interpretability, SHAP (SHapley Additive exPlanations) analysis was conducted on the best-performing model (XGBoost). In the test set, the following metrics were calculated for each model: AUC (macro-averaged across classes), overall accuracy, Cohen’s kappa coefficient, macro-averaged F1-score, sensitivity (recall), and specificity. Details are available in Table 4: Predictive Performance of Machine Learning Models, Figure 7 the predictive performance of six different models.

**Table 4:** Predictive performance of machine learning models.

Model	AUC	Accuracy	Kappa	F1-score	Sensitivity	Specificity
LOG	0.83	0.91	0.8	0.76	0.79	0.97
XGBoost	0.83	0.91	0.8	0.78	0.8	0.96
SVM	0.8	0.84	0.68	0.67	0.72	0.95
AdaBoost	0.8	0.87	0.71	0.66	0.66	0.93
GBM	0.75	0.88	0.74	0.72	0.72	0.94
RF	0.79	0.9	0.78	0.71	0.71	0.95

### SHAP-based model interpretation

**Figure 8:**
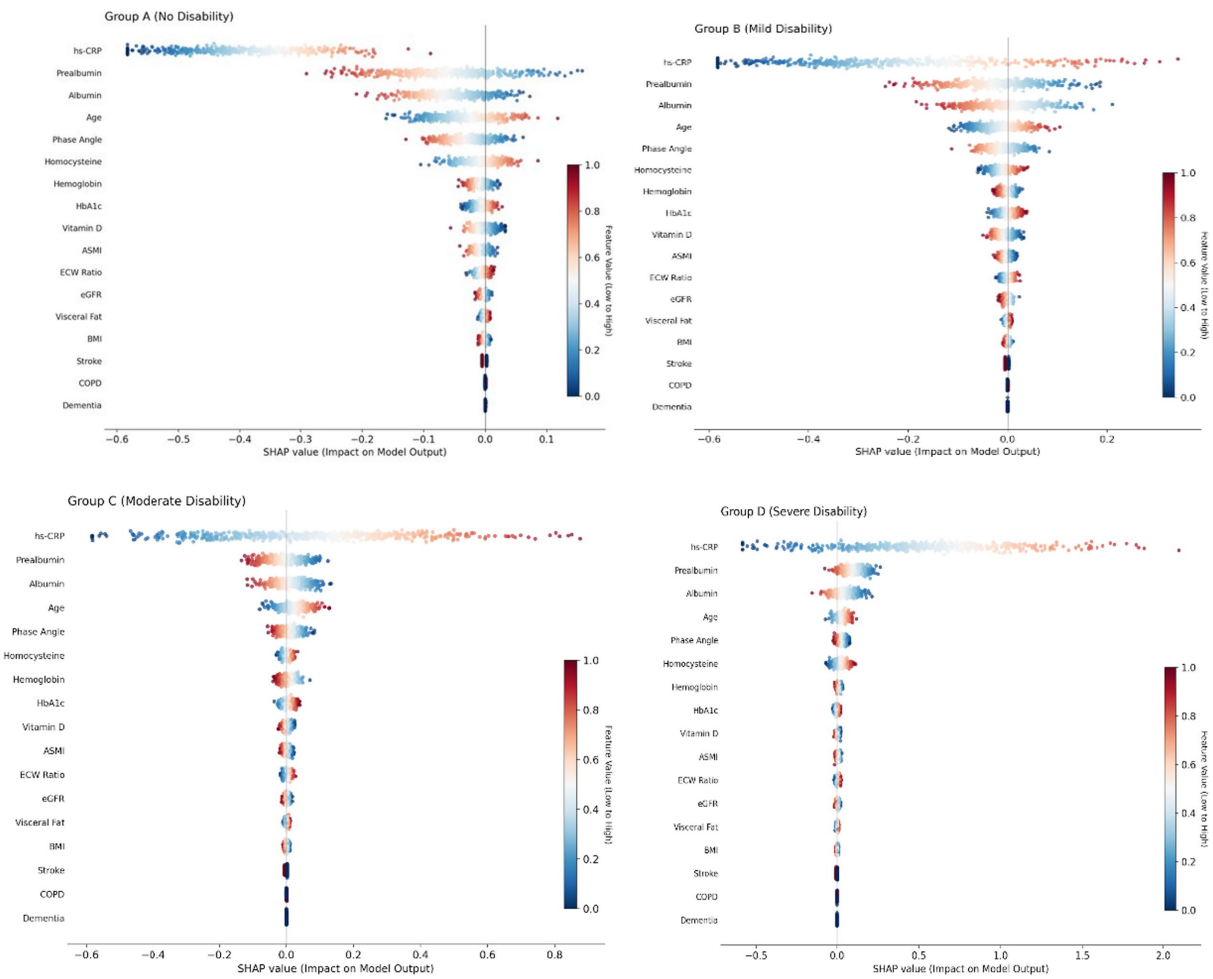
Model interpretation and feature contribution analysis via SHAP composed of four subplots, the figure displays the marginal contributions of 17 core predictive variables to the prediction of each disability level. Each row in the figure represents a feature, the horizontal axis represents the SHAP value, and the color intensity represents the relative level of the feature value (red for high values, blue for low values). Inflammatory-Nutritional Axis: In all disability levels, high-sensitivity C-reactive protein (hs-CRP), prealbumin, and albumin all showed extremely high predictive weights. High values of hs-CRP (red) were significantly associated with positive SHAP values in Group C and Group D, indicating that systemic inflammation is a core risk factor driving severe disability. Body composition indicators: Whole-body phase angle (PhA) and appendicular skeletal muscle index (ASMI) are the strongest protective factors for predicting no disability (Group A).

Considering the ordinal nature of disability severity (none < mild < moderate < severe), we treated the results as ordinal variables. A tree-based regression model (XGBoost) was used to predict the severity score (mapped from 0 to 3). Therefore, a SHAP value was calculated for each feature, representing its contribution to the progression of disability severity. Positive SHAP values indicate a contribution towards more severe disease (groups C/D), while negative values indicate a protective effect (group A). This is illustrated in Figure 8. Model Interpretation and Feature Contribution Analysis via SHAP.

## Discussion

This study developed and validated a multi-class machine learning model using EMR data from 523 older adults inpatients to predict four distinct levels of disability. By integrating 17 core clinical and laboratory predictors selected via LASSO regression, we achieved high predictive accuracy and provided robust interpretability through SHAP analysis.

### Model performance and algorithmic comparison

Among the six evaluated algorithms, Logistic Regression (LOG) and XGBoost demonstrated superior predictive performance, both achieving an AUC of 0.83 and an accuracy of 0.91. The high performance of LOG suggests that while functional decline is a complex process, many clinical predictors maintain a stable linear relationship with disability levels. However, XGBoost outperformed other models in terms of sensitivity (0.80) and F1-score (0.78), indicating its robustness in identifying minority classes, such as moderate and severe disability (Groups C and D). This advantage is crucial for clinical practice, where the early identification of patients at the highest risk of functional loss is the primary goal of intervention.

### Biological plausibility of identified predictors

The SHAP interpretation revealed that the model’s decisions were driven by three primary clusters of features: neurological status, body composition, and the “inflammation-nutrition-edema” cycle.

Neurological Dominance: As expected, stroke and dementia syndrome exhibited the highest Mean |SHAP| values, identifying them as the most potent drivers of severe disability. This aligns with previous evidence that irreversible neurological damage is the leading contributor to Disability-Adjusted Life Years (DALYs) globally. The Sarcopenia Axis: Beyond specific diseases, Appendicular Skeletal Muscle Index (ASMI) and Whole-body Phase Angle (PhA) showed significant predictive weight. The negative SHAP values for ASMI indicate that maintaining skeletal muscle mass is a foundational physiological defense against functional decline. Notably, PhA emerged as a critical biomarker of cell membrane integrity and cellular vitality, with its predictive importance surpassing that of traditional BMI. This suggests that clinical assessments should shift from “weight-centric” to “quality-centric” monitoring of muscle function.

The Inflammation-Nutrition-Edema Cycle: The model identified a synergistic relationship between high hs-CRP, low albumin/prealbumin, and a high extracellular water (ECW) ratio. Chronic systemic inflammation (elevated hs-CRP) accelerates protein consumption (malnutrition), leading to altered cell membrane permeability and increased ECW ratio. Our findings suggest that an elevated ECW/TBW ratio may serve as an early, sensitive warning signal for frailty and functional deterioration in hospitalized older adults patients.

### Clinical implications

These findings offer several practical insights for geriatric care: Automated Risk Stratification: The XGBoost model can be integrated into hospital information systems to automatically screen for patients at high risk of moderate-to-severe disability based on routine EMR data, triggering immediate comprehensive geriatric assessments (CGA). Targeted Therapeutic Interventions: The identification of modifiable risk factors—such as nutritional status (Albumin, Vitamin D) and muscle mass (ASMI)—provides specific targets for multidisciplinary interventions, including resistance training and nutritional supplementation, to delay the progression of disability. Resource Allocation: Accurate four-level grading assists in planning rehabilitation intensity and post-discharge care trajectories (e.g., home care vs. long-term care facilities), thereby optimizing the efficiency of the healthcare system in an aging society.

### Strengths and limitations

The strengths of this study include the use of multi-dimensional Real-World Data (RWD) from two tertiary hospitals, the systematic comparison of six mainstream algorithms, and the use of SHAP for transparent clinical interpretability. However, some limitations must be acknowledged. First, the retrospective nature and single-region focus of the study necessitate future external validation in diverse populations. Second, the EMR data lacked certain psychosocial and environmental factors, such as social support and economic status, which are known to influence disability. Finally, the cross-sectional nature of the disability assessment limits the model’s ability to reflect dynamic transitions in functional status over time.

Although the sample size (*n* = 523) may appear modest for machine learning applications, it remains comparable to several prior clinical prediction studies using structured hospital datasets. Furthermore, the model was developed using structured clinical variables with relatively low dimensionality, which reduces the risk of overfitting. Nevertheless, future studies with larger multicenter datasets will be necessary to further validate the robustness of the proposed model. Another limitation of the present study is the lack of external validation. The current model was developed using inpatient data from hospitals in Dalian and Shanghai, which may limit the generalizability of the findings. Future studies should validate the model using independent datasets from other regions and healthcare systems to confirm its robustness and clinical applicability.

## Conclusion

This study developed and validated a machine learning-based four-level disability prediction model using EMR data from two tertiary hospitals in Northeast China. By employing LASSO regression for feature selection, we identified 17 clinically meaningful predictors that are robustly associated with the severity of functional impairment. Our results demonstrate that Logistic Regression and XGBoost are the most effective models for this task, with XGBoost showing superior performance in sensitivity and providing enhanced clinical interpretability through SHAP analysis.

The findings confirm the feasibility and clinical utility of using routine EMR data to automate disability risk assessment in older adults inpatients. Such models can facilitate early risk identification, optimize the allocation of healthcare resources, and support the development of targeted multidisciplinary intervention strategies. Future research should focus on external validation in diverse geographical regions and the exploration of longitudinal EMR data to predict dynamic disability trajectories and evaluate the impact of model-driven interventions in real-world clinical settings.

## Data Availability

The datasets presented in this article are not readily available due to data confidentiality restrictions. Requests to access the datasets should be directed to 229280127@qq.com.
