# Supplementary material for: Assessment of disability in the older adults using electronic health record–based data: a machine learning approach
Source: Front Public Health. 2026 May 1;14:1823727. doi: 10.3389/fpubh.2026.1823727 (PMC13176208; doi:10.3389/fpubh.2026.1823727)
Supplement: Supplementary file 1 [file Table_1.docx]

Supplementary Table 1 primary disease categories.

| No. | disease categories | A | B | C | D |
| --- | --- | --- | --- | --- | --- |
| 0 | None / No underlying disease | 13（3.9%） | 2（1.6%） | 0（0.0%） | 0（0.0%） |
| 1 | Coronary atherosclerotic heart disease | 11（3.3%） | 6（4.9%） | 4（10.0%） | 3（10.0%） |
| 2 | Chronic obstructive pneumonia | 60（18.2%） | 15（12.2%） | 10（25.0%） | 11（36.7%） |
| 3 | Stroke | 109（33.0%） | 59（47.9%） | 5（12.5%） | 3（10.0%） |
| 4 | Lower limb fracture | 54（16.4%） | 23（18.7%） | 9（22.5%） | 6（20.0%） |
| 5 | Parkinson’s disease | 2（0.6%） | 4（3.3%） | 1（2.5%） | 2（6.7%） |
| 6 | Dementia syndrome | 4（1.2%） | 2（1.6%） | 1（2.5%） | 3（10.0%） |
| 7 | Pneumonia | 7（2.1%） | 2（1.6%） | 1（2.5%） | 0（0.0%） |
| 8 | Type 2 diabetes mellitus | 22（6.7%） | 2（1.6%） | 5（12.5%） | 1（3.3%） |
| 9 | Osteoporosis | 36（10.9%） | 3（2.4%） | 1（2.5%） | 1（3.3%） |
| 10 | Hypertension | 6（1.8%） | 4（3.3%） | 1（2.5%） | 1（3.3%） |
| 11 | Arthritis | 5（1.5%） | 1（0.8%） | 1（2.5%） | 1（3.3%） |
| 12 | Malignant tumors | 1（0.3%） | 1（0.8%） | 1（2.5%） | 0（0.0%） |
| 13 | Others | 2（0.6%） | 1（0.8%） | 0（0.0%） | 0（0.0%） |

Supplementary Table 2 Socio-demographic characteristics,

|  | A (N=330) | B (N=123) | C (N=40) | D (N=30) |
| --- | --- | --- | --- | --- |
| Height (cm) | |  |  |  |
| Mean (SD) | 161.5 (7.2) | 161.2 (7.0) | 160.9 (6.8) | 157.0 (6.5) |
| Median [Min, Max] | 161.0 [145.0, 180.0] | 161.0 [145.0, 178.0] | 160.5 [144.0, 176.0] | 157.0 [138.0, 170.0] |
| Weight (kg) | |  |  |  |
| Mean (SD) | 62.8 (9.4) | 62.2 (9.2) | 61.5 (9.0) | 54.2 (8.2) |
| Median [Min, Max] | 62.5 [45.0, 85.0] | 62.0 [44.0, 83.0] | 61.0 [43.0, 82.0] | 54.0 [38.0, 74.0] |
| Body Mass Index (kg/m²) | | |  |  |
| Mean (SD) | 24.0 (3.0) | 23.9 (2.9) | 23.7 (2.8) | 21.9 (2.7) |
| Median [Min, Max] | 23.8 [18.0, 30.5] | 23.6 [17.8, 30.0] | 23.4 [17.5, 29.5] | 21.8 [15.0, 27.5] |
| Gender: |  |  |  |  |
| 1 - Male | 172（52.1%） | 49（39.8%） | 16（40.0%） | 11（38.3%） |
| 2 - Female | 158（47.9%） | 74（60.2%） | 24（60.0%） | 19（61.7%） |
| Age (Years) |  |  |  |  |
| Mean (SD) | 68.0 (6.6) | 71.5 (6.2) | 78.5 (5.7) | 80.2 (4.3) |
| Median [Min, Max] | 67.0 [60.0, 93.0] | 71.0 [60.0, 86.0] | 79.5 [70.0, 85.0] | 79.0 [74.0, 86.0] |
| Education |  |  |  |  |
| 1-Illiterate | 17（5.0%） | 10（8.0%） | 5（12.0%） | 5（15.0%） |
| 2-Primary | 66（20.0%） | 37（30.1%） | 14（35.0%） | 12（40.0%） |
| 3-Middle/High School | 134（40.6%） | 55（44.7%） | 15（38.0%） | 11（35.0%） |
| 4-Junior College or Above | 113（34.4%） | 21（17.2%） | 6（15.0%） | 3（10.0%） |

Supplementary Table 3 Body composition-related indicators

| Variable | Group A | Group B | Group C | Group D |
| --- | --- | --- | --- | --- |
| ASMI (kg/m²) | |  |  |  |
| Mean (SD) | 7.25 (0.85) | 6.85 (0.80) | 6.40 (0.75) | 5.95 (0.70) |
| Median [Min, Max] | 7.20 [5.9, 9.2] | 6.80 [5.5, 8.6] | 6.30 [5.0, 7.8] | 5.90 [4.8, 7.2] |
| Total Body Water (L) | |  |  |  |
| Mean (SD) | 33.5 (3.2) | 32.6 (3.3) | 32.0 (3.4) | 32.1 (3.2) |
| Median [Min, Max] | 33.4 [24.0, 41.2] | 32.5 [23.5, 40.0] | 31.8 [22.8, 39.5] | 31.4 [22.0, 38.0] |
| Muscle Mass (kg) | |  |  |  |
| Mean (SD) | 25.6 (3.1) | 23.9 (3.0) | 22.1 (2.9) | 20.4 (2.8) |
| Median [Min, Max] | 25.5 [18.0, 33.5] | 23.8 [16.5, 31.5] | 22.0 [15.0, 29.8] | 20.2 [14.0, 28.0] |
| Fat-free Mass (kg) | |  |  |  |
| Mean (SD) | 46.4 (5.0) | 45.2 (5.1) | 44.3 (4.9) | 44.3 (4.7) |
| Median [Min, Max] | 48.2 [35.0, 60.0] | 47.0 [34.0, 58.0] | 45.8 [33.0, 56.5] | 44.9 [32.5, 55.0] |
| Protein (kg) | |  |  |  |
| Mean (SD) | 9.2 (1.1) | 9.0 (1.2) | 8.8 (1.2) | 8.7 (1.1) |
| Median [Min, Max] | 9.1 [6.5, 12.0] | 8.9 [6.3, 11.8] | 8.7 [6.0, 11.5] | 8.6 [5.8, 11.0] |
| Minerals (kg) | |  |  |  |
| Mean (SD) | 3.7 (0.4) | 3.6 (0.4) | 3.5 (0.4) | 3.5 (0.4) |
| Median [Min, Max] | 3.6 [2.8, 4.6] | 3.6 [2.7, 4.5] | 3.5 [2.6, 4.4] | 3.5 [2.6, 4.2] |
| Skeletal Muscle Mass (kg) | | |  |  |
| Mean (SD) | 23.8 (3.5) | 22.1 (3.2) | 20.4 (3.0) | 18.9 (2.8) |
| Median [Min, Max] | 23.6 [16.5, 33.2] | 22.0 [15.0, 30.0] | 20.2 [13.8, 28.0] | 18.7 [12.5, 25.5] |
| Body Fat Percentage (%) | |  |  |  |
| Mean (SD) | 25.5 (6.2) | 28.2 (6.8) | 30.6 (7.0) | 32.0 (7.5) |
| Median [Min, Max] | 25.2 [12.0, 40.0] | 28.0 [14.0, 42.5] | 30.5 [15.0, 45.5] | 33.4 [17.0, 47.0] |
| Intracellular Water (L) | |  |  |  |
| Mean (SD) | 21.5 (2.5) | 21.1 (2.6) | 20.8 (2.6) | 20.5 (2.5) |
| Median [Min, Max] | 21.4 [14.0, 27.0] | 21.0 [13.5, 26.5] | 20.7 [13.0, 26.0] | 20.4 [12.8, 25.5] |
| Extracellular Water (L) | |  |  |  |
| Mean (SD) | 12.0 (1.5) | 11.5 (1.6) | 11.2 (1.7) | 11.6 (1.6) |
| Median [Min, Max] | 11.9 [8.0, 16.0] | 11.8 [7.8, 15.5] | 11.6 [7.5, 15.0] | 11.5 [7.3, 14.5] |
| Extracellular Water Ratio (%) | 38.2 (1.5) | 39.0 (1.6) | 39.8 (1.7) | 40.5 (1.8) |
| Median [Min, Max] | 38.1 [35.5, 41.5] | 39.0 [36.0, 42.5] | 39.9 [37.0, 43.0] | 40.6 [37.5, 44.0] |
| Visceral Fat Area (cm²) | |  |  |  |
| Mean (SD) | 95.5 (28.0) | 105.0 (30.5) | 115.0 (32.1) | 118.0 (34.2) |
| Median [Min, Max] | 94.0 [45.0, 180.0] | 108.0 [50.0, 190.0] | 121.0 [55.0, 200.0] | 134.0 [60.0, 210.0] |
| Basal Metabolic Rate (kcal) | | |  |  |
| Mean (SD) | 1450 (160) | 1370 (150) | 1290 (140) | 1210 (130) |
| Median [Min, Max] | 1440 [1100, 1800] | 1360 [1000, 1700] | 1280 [950, 1650] | 1200 [900, 1600] |
| Whole-body Phase Angle (°) | | |  |  |
| Mean (SD) | 6.1 (0.9) | 5.5 (0.8) | 4.9 (0.7) | 4.3 (0.6) |
| Median [Min, Max] | 6.1 [4.0, 8.5] | 5.4 [3.8, 7.5] | 4.8 [3.5, 6.8] | 4.2 [3.2, 6.0] |

Supplementary Table 4 Laboratory indicators

| Variable | Group A | Group B | Group C | Group D |
| --- | --- | --- | --- | --- |
| hs-CRP (mg/L) | |  |  |  |
| Mean (SD) | 1.5 (0.8) | 2.8 (1.5) | 5.2 (2.1) | 8.5 (3.5) |
| Median [Min, Max] | 1.4 [0.2, 5.0] | 2.5 [0.3, 8.5] | 4.8 [1.2, 12.0] | 7.9 [2.5, 18.0] |
| Hemoglobin (g/L) | |  |  |  |
| Mean (SD) | 139 (17.4) | 126 (18.7) | 116 (27.7) | 105 (15.2) |
| Median [Min, Max] | 139 [85, 184] | 125 [69, 163] | 111 [69, 162] | 104 [65, 145] |
| eGFR |  |  |  |  |
| Mean (SD) | 84.6 (10.5) | 83.2 (13.6) | 75.4 (12.1) | 68.5 (15.3) |
| Median [Min, Max] | 90.0 [55, 120] | 87.5 [45, 110] | 76.0 [35, 95] | 67.0 [30, 90] |
| Total Cholesterol (mmol/L) | | |  |  |
| Mean (SD) | 4.95 (1.19) | 4.77 (1.20) | 5.06 (1.12) | 4.20 (1.05) |
| Median [Min, Max] | 4.96 [1.97, 10.6] | 4.44 [1.72, 8.05] | 5.05 [4.23, 9.46] | 4.15 [2.5, 7.5] |
| Triglycerides (mmol/L) | |  |  |  |
| Mean (SD) | 1.64 (0.99) | 1.83 (1.05) | 2.10 (1.20) | 1.55 (0.85) |
| Median [Min, Max] | 1.36 [0.49, 7.67] | 1.61 [0.62, 5.85] | 1.85 [0.80, 6.50] | 1.40 [0.5, 4.5] |
| HDL-C (mmol/L) | |  |  |  |
| Mean (SD) | 1.23 (0.26) | 1.12 (0.28) | 0.99 (0.14) | 1.05 (0.15) |
| Median [Min, Max] | 1.23 [0.59, 2.38] | 1.12 [0.61, 1.80] | 1.01 [0.76, 1.25] | 1.03 [0.70, 1.45] |
| LDL-C (mmol/L) | |  |  |  |
| Mean (SD) | 2.85 (0.93) | 2.69 (0.99) | 2.72 (0.41) | 2.45 (0.55) |
| Median [Min, Max] | 2.87 [0.78, 7.51] | 2.63 [0.11, 4.99] | 2.59 [2.27, 3.45] | 2.40 [1.2, 4.0] |
| Albumin (g/L) | |  |  |  |
| Mean (SD) | 42.5 (4.4) | 38.2 (6.8) | 35.9 (3.7) | 32.1 (5.2) |
| Median [Min, Max] | 43.0 [28, 55] | 39.1 [25, 49] | 35.7 [23, 45] | 32.5 [20, 42] |
| Prealbumin (mg/L) | |  |  |  |
| Mean (SD) | 287 (41.9) | 264 (41.2) | 252 (23.7) | 195 (30.5) |
| Median [Min, Max] | 288 [150, 452] | 261 [152, 387] | 255 [207, 278] | 190 [100, 260] |
| ALT (U/L) |  |  |  |  |
| Mean (SD) | 22.5 (12.5) | 22.6 (15.4) | 18.5 (10.6) | 17.6 (5.8) |
| Median [Min, Max] | 19.5 [5, 85] | 17.7 [7, 89] | 15.5 [7, 41] | 16.9 [10, 38] |
| AST (U/L) |  |  |  |  |
| Mean (SD) | 24.5 (10.5) | 21.2 (8.2) | 18.5 (6.5) | 19.0 (4.5) |
| Median [Min, Max] | 22.0 [10, 75] | 20.1 [10, 66] | 17.5 [12, 45] | 18.7 [15, 38] |
| Fasting Blood Glucose (mmol/L) | | |  |  |
| Mean (SD) | 7.23 (2.77) | 8.45 (4.02) | 9.29 (5.28) | 7.77 (3.01) |
| Median [Min, Max] | 6.34 [3.7, 18.0] | 6.92 [4.1, 24.9] | 6.90 [5.1, 20.0] | 6.37 [5.0, 17.2] |
| HbA1c (%) |  |  |  |  |
| Mean (SD) | 6.5 (1.5) | 7.92 (1.80) | 8.16 (1.74) | 7.50 (1.20) |
| Median [Min, Max] | 6.1 [4.5, 12.0] | 7.30 [5.9, 13.5] | 8.20 [6.4, 11.5] | 7.40 [5.4, 10.8] |
| Vitamin D (ng/mL) | |  |  |  |
| Mean (SD) | 20.4 (6.9) | 22.8 (7.5) | 18.5 (5.2) | 15.2 (4.1) |
| Median [Min, Max] | 20.3 [6.4, 55] | 21.8 [8.3, 55.8] | 18.0 [8, 35] | 14.5 [5, 28] |
| BUN (mmol/L) | |  |  |  |
| Mean (SD) | 5.80 (2.10) | 6.85 (3.09) | 7.90 (2.50) | 9.20 (3.10) |
| Median [Min, Max] | 5.50 [2.5, 12.0] | 6.10 [2.6, 18.1] | 7.50 [3.5, 15.0] | 8.80 [4.0, 20.0] |
